# Religiosity and Health Outcomes in a Cohort of Romanian Patients Hospitalized for COVID-19

**DOI:** 10.1007/s10943-024-02120-6

**Published:** 2024-09-10

**Authors:** Stefan Frent, Alexandru-Filip Popovici, Adrian Balan, Bianca Cerbu, Iosif Marincu, Stefan Mihaicuta, Andras Bikov

**Affiliations:** 1 Center for Research and Innovation in Precision Medicine of Respiratory Diseases, Department of Pulmonology, “Victor Babes” University of Medicine and Pharmacy Timisoara, Eftimie Murgu Sq. No. 2, 300041 Timisoara, Romania; 2 Faculty of Psychology and Educational Sciences, University of Bucharest, Panduri Street No. 90, 050657 Bucharest, Romania; 3 Victor Babes” University of Medicine and Pharmacy Timisoara, Eftimie Murgu Sq. No. 2, 300041 Timisoara, Romania; 4 Infectious Diseases Department, “Victor Babes” University of Medicine and Pharmacy Timisoara, Eftimie Murgu Sq. No. 2, 300041 Timisoara, Romania; 5 Wythenshawe Hospital, Manchester University NHS Foundation Trust, Manchester Academic Health Science Centre, Oxford Road, Manchester, M13 9WL UK; 6 Division of Infection, Immunity & Respiratory Medicine, Faculty of Biology, Medicine and Health, University of Manchester, Manchester, M13 9PT UK

**Keywords:** COVID-19, Hospitalization, Religiosity, Mortality

## Abstract

There is a growing body of evidence for the interrelation between health status and religious beliefs. Our aim was to evaluate the level of religiosity in patients hospitalized for COVID-19 and to assess the link between religiosity and measurable health outcomes. This was an observational, single-center study which included patients with moderate-to-severe forms of COVID-19. A total of 112 patients were enrolled in the study, of whom 77 were highly religious (CRS-15 score ≥ 4) and 35 non-highly religious (CRS-15 score < 4). There was no difference in demographics or prevalence of comorbidities between the two groups. Furthermore, we found no difference between groups in radiological extension of lung lesions, length of hospital stays, or ICU need; however, in-hospital mortality rate was significantly lower in highly religious group (1% vs. 14%, *p* = 0.005). Serum ferritin level at admission was significantly lower (*p* = 0.03) and prevalence of post-COVID-19 pulmonary sequelae significantly higher in highly religious group (*p* = 0.02).

## Introduction

There is a growing body of evidence for the interrelation between health status and religious beliefs (Diego-Cordero et al., 2022). Coping strategies are very important when fighting life-threatening conditions.

Religiosity can be defined as the beliefs, values, practices, and rituals of a particular faith. Spirituality is distinct from religiosity as it addresses ultimate questions about life’s meaning, is more subjective, and is less visible and measurable than religiosity (Green et al., 2011). Several studies have analyzed the role of religiosity/spirituality as a coping mechanism in patients with chronic respiratory conditions such as asthma (Adeli et al., 2014), emphysema (Green et al., 2011), COPD (da Silva et al., 2018), in patients with chronic debilitating conditions, such as chronic pain (Büssing et al., 2009), or in patients with fatal diseases such as advanced cancer (Tarakeshwar et al., 2006). The results from most of these studies demonstrate that outcomes are better in patients utilizing positive religious coping mechanisms.

### The Need for Testing this Hypothesis

According to the latest available census data (2021), Romanians declare themselves religious in a vast majority (99%) (Institutul National de Statistica, 2023). However, to our knowledge, there is no study on the interrelation between religious beliefs and health status or disease state in the Romanian population.

Although several studies have been published on religious coping mechanisms and the role of prayer in the fight with a disease state, most of these studies focused mainly on patients with chronic conditions. Further research is also needed for a better understanding of the complex interrelations between the human body and soul, leading to a better understanding of human nature.

The human nature is far too complex to be studied from a single perspective; therefore, a combined multidisciplinary approach (i.e., scientific, theological, and philosophical) is required to deliver a scientifically sound research project on this interesting and controversial topic. Spirituality/religiosity plays a special role in times of distress, such as wars, natural disasters, or a pandemic-induced global crisis (Marshall & Smith, 2015; Smith et al., 2009).

The COVID-19 pandemic caused by severe acute respiratory syndrome coronavirus 2 (SARS-CoV-2), which first emerged in Wuhan, China, in December 2019, was probably the biggest challenge in the last hundred years for health-care systems worldwide, and one of the deadliest pandemics in history, with more than 648 million cases and 6.65 million confirmed deaths, as of December 2022 (Coronavirus Resource Center, 2022). The current pandemic has also triggered serious social and economic disruption around the world, including a global recession. The impact of this tremendous burden on mental health cannot be overestimated (Frent et al., 2022). In this context, spirituality and religiosity have emerged as valuable resilience resources. Targeting positive beliefs, religious activities, as well as religious social support, served as coping mechanisms to overcome physical and mental health problems, alleviating stress, and psychological suffering (Chirico & Nucera, 2020).

## Objective

The aim of the current study was to evaluate the level of religiosity in a Romanian cohort of patients hospitalized for COVID-19 using a validated tool and to assess correlations between religiosity and measurable health outcomes such as: survival, number of hospitalized days, and occurrence of complications or sequelae.

## Methods

### Study Design

This was an observational, single-center study which included patients with moderate-to-severe forms of COVID-19 requiring hospitalization. Inclusion criteria were as follows: patients hospitalized or with a documented history of hospitalization for COVID-19, willingness, and ability to complete a religiosity questionnaire. We excluded patients with COVID-19 who did not require or were not hospitalized, patients with a history of hospitalization for COVID-19 but who were not able to provide medical documents (e.g., discharge letter), critically ill patients, patients with altered state of consciousness, and those unable to complete the questionnaire. All patients enrolled in the study provided a signed informed consent form. The study protocol was approved by the Ethics Committee of “Victor Babes” Infectious Diseases and Pulmonology Clinical Hospital Timisoara, Romania (11,827/2021), and the research was conducted in accordance with the principles of the Declaration of Helsinki.

### Assessment of Religiosity Level

Patients’ religiosity was assessed using the Centrality of Religiosity Scale (CRS-15). The English version of CRS-15 was translated into Romanian using the forward–backward translation design and following the guidelines provided by the literature (Beaton et al., 2000; Dunn et al., 1994). The scale was originally developed by Huber and Huber (2012) to measure the centrality, importance, or salience of religious meanings in personality. The Romanian version of the scale was derived from the English version and was validated by the author (Huza, 2018). The items were formulated using simple and appropriate language relative to the concept. The scale consisted of 15 items divided into five subscales: intellect (1, 6, 11)—SS1, ideology (2, 7, 12)—SS2, public practice (3, 8, 13)—SS3, private practice (4, 9, 14)—SS4, and religious experience (5, 10, 15)—SS5. Each subscale contained three items that measured the objective or subjective frequency or the intensity of personal religious constructs.

Answers were measured by a five-point Likert scale, with the exception of certain items that had a different coding system. For events that may not occur regularly, subjective frequencies were recorded in five levels (never, rarely, occasionally, often, and very often). For events where the frequency had an insignificant role (e.g., belief in something divine), the intensity or importance was evaluated as: not at all, not very much, moderately, quite a bit, or very much so. The item that refers to participation in religious service was coded as follows: more than once week and once a week—5, one to three times a month—4, a few times a year—3, less often—2, and never—1. The item that refers to the objective frequency of prayer was coded as follows: several times a day and once a day—5, more than once a week—4, once a week and one to three times a month—3, a few times a year or less—2, and never—1. The subscale results were the average of the items. The total result (CRS-15) was the sum of the subscale’s scores divided through the number of scored items. This allowed for a range of the CRS-15 total score between 1.0 and 5.0. For the categorization of the groups, Huber and Huber (2012) proposed three thresholds: 1.0–2.0 for “not religious,” 2.1–3.9 for “religious,” and 4.0–5.0 for “highly religious” (Huber & Huber, 2012).

The Cronbach alpha for the CRS-15 was 0.93. For the subscales, Cronbach’s alpha was as follows: 0.80 for intellect (*M* = 3.20; SD = 0.96), 0.61 for ideology (*M* = 4.12; SD = 0.90), 0.82 for public practice (*M* = 3.64; SD = 1.16), 0.85 for private practice (*M* = 3.96; SD = 1.11), and 0.87 for religious experience (*M* = 3.44; SD = 1.13).

The CRS-15 religiosity questionnaire was offered for completion to consecutive COVID-19 patients who satisfied inclusion/exclusion criteria between November 2021 and March 2022, either during their hospitalization for the acute phase of the disease, during the rehabilitation phase, or at the follow-up evaluation.

### Health Outcomes Measures

Medical data related to the SARS-CoV-2 infection episode for patients who completed the religiosity questionnaire were retrieved from the hospital’s electronic database, or from relevant medical documents provided by the patients (i.e., discharge letter), as recorded by the treating physicians. We collected demographic data, COVID-19 vaccination status, COVID-19 symptoms reported at hospital admission, comorbidities, laboratory data, the radiological extension of lung lesions, treatment, vital status at the end of hospitalization period, number of hospitalized days, and the ICU need.

Additionally, we collected post-COVID-19 follow-up data, including vital status, clinical evolution, the presence of pulmonary sequelae on the chest CT scan, and occurrence of post-COVID-19 complications. The latter were defined as persistent symptoms or new conditions diagnosed after the initial episode of SARS-CoV-2 infection and recorded upon follow-up evaluation. All patients were routinely recommended to attend a follow-up assessment at 1–3 months post-discharge, comprising a clinical evaluation, a chest CT scan, laboratory tests, and lung function measurement. A follow-up phone contact was attempted for those who did not attend the follow-up evaluation in order to collect information on the vital status and post-COVID-19 clinical evolution.

### Statistical Analysis

We used the JASP 0.11.1 (Amsterdam, Netherlands) software for statistical analysis. Based on their CRS-15 score, patients were divided into highly religious (≥ 4.0) and non-highly religious (< 4.0) groups. Demographics, symptoms, and the prevalence of comorbidities were compared between the two groups with Mann–Whitney and Chi-square tests. Mortality, hospital stay, ICU need, laboratory results, and post-COVID outcomes were compared between the two groups with ANCOVA adjusted for age, gender, vaccination status, radiological extension of lung lesions, and dexamethasone use. The correlation between CRS-15 score and laboratory values was assessed with the Spearman’s test. Data are expressed as mean ± standard deviation or percentages. A *p* value < 0.05 was considered significant.

## Results

One hundred and forty-eight patients with confirmed SARS-CoV-2 infections were screened, of whom eight patients declined the study participants. One hundred and twelve consecutive patients who satisfied the inclusion and exclusion criteria and had available SARS-CoV-2 infection-related medical records were enrolled in the study and completed the CRS-15 questionnaire. According to their CRS-15 score, 77 patients (68.75%) were assessed as highly religious (≥ 4.0), while the other 35 (31.25%) comprised the control group of non-highly religious.

### Patients’ Characteristics

There was no difference in demographics or prevalence of comorbidities between the two groups. Of note, there was a tendency for higher prevalence of females in the highly religious group (Table 1).

**Table 1:** Patients’ characteristics

	Non-highly religious (*n* = 35)	Highly religious (*n* = 77)	*P* value
Age (years)	60 ± 12	64 ± 10	0.12
Gender (males %)	68	49	0.06
Vaccinated (%)	46	30	0.31
Obesity (%)	14	20	0.51
Hypertension (%)	49	56	0.47
Atrial fibrillation (%)	9	4	0.31
Chronic heart failure (%)	6	5	0.91
Peripheral vascular disease (%)	1	6	0.18
Dyslipidemia (%)	3	1	0.56
Asthma (%)	11	10	0.87

### Patients’ Symptoms

Comparing the two groups, symptoms at admission to hospital were similar. Of note, highly religious patients more often complained about chills at presentation (Table 2).

**Table 2:** Patients’ symptoms

	Non-highly religious (*n* = 35)	Highly religious (*n* = 77)	*P* value
Dyspnea (%)	77	61	0.09
Anosmia (%)	11	12	0.97
Ageusia (%)	3	6	0.43
Anorexia (%)	20	34	0.14
Nausea (%)	11	19	0.29
Vomiting (%)	9	13	0.50
Odynophagia (%)	3	3	0.94
Fever (%)	49	52	0.74
Chills (%)	9	29	0.02
Sweats (%)	6	8	0.69
Dry cough (%)	40	42	0.88
Productive cough (%)	23	27	0.61
Arthralgia (%)	6	9	0.54
Myalgia (%)	11	16	0.56
Chest pain (%)	3	4	0.78
Headache (%)	14	21	0.41
Somnolence (%)	3	3	0.94
Fatigue (%)	54	51	0.72
Asthenia (%)	34	39	0.64
Diarrhea (%)	11	8	0.53
Weight loss (%)	3	4	0.78
Vertigo (%)	9	4	0.31

### Laboratory Parameters

The blood ferritin level at hospital admission was significantly lower in the highly religious group. No other differences between groups were noticed in the laboratory parameters assessed (Table 3).

**Table 3:** Laboratory parameters

	Non-highly religious (*n* = 35)	Highly religious (*n* = 77)	*P* value*
White blood cell count (*1000/μL)	8.8 ± 5.8	7.6 ± 4.1	0.86
Hemoglobin (g/dL)	135 ± 16	135 ± 17	0.44
Platelets (*1000/μL)	274 ± 130	265 ± 116	0.25
Neutrophils (%)	77 ± 14	76 ± 11	0.98
Eosinophils (%)	0.4 ± 1.5	0.3 ± 0.8	0.10
Basophils (%)	0.1 ± 0.3	0.1 ± 0.1	0.06
Lymphocytes (%)	15 ± 10	16 ± 10	0.87
Ferritin (μg/L)	1960 ± 1336	1082 ± 852	0.03
CRP (mg/L)	111 ± 83	97 ± 88	0.75

*Adjusted for age, gender, vaccination status, radiological extension, and dexamethasone use

Analyzing the relationship between CRS-15 total and subscale scores as well as laboratory parameters, we observed a significant correlation between SS1 and ferritin (ρ = − 0.367, *p* < 0.01) as well as CRP (ρ = − 0.200, *p* = 0.039), between SS2 and eosinophils (ρ = − 0.206, *p* = 0.03) as well as basophils (ρ = − 0.291, *p* < 0.01), between SS5 and ferritin (ρ = − 0.300, *p* < 0.01), and between CRS-15 total score and ferritin (ρ = − 0.307, *p* = 0.002).

### Chest Imaging

In the non-highly religious group, 8% had no, 23% had mild (0–25%), 46% had moderate (25–50%), and 23% had severe (> 50%) radiological involvement. In the highly religious group, the corresponding numbers were 1%, 27%, 46%, and 26%. There was no difference between the two groups (p = 0.27).

### COVID-19-Related Health Outcomes

Eighty-six percent (86%) in both groups were treated with dexamethasone (*p* = 1.0), antiviral treatment was given to 89% and 88% (non-highly religious and highly religious, respectively, *p* = 0.97). More patients have ultimately died in the non-highly religious group (14% vs. 1%, *p* < 0.01); however, following adjustment for age, gender, vaccination status, radiological extension, and dexamethasone treatment, there was only a tendency for the observed difference (*p* = 0.08). There was no difference in the lengths of hospital stay (17 ± 10 vs. 17 ± 10 days, unadjusted *p* = 0.989, adjusted *p* = 0.300) or ICU need (11% vs. 6%, unadjusted *p* = 0.378, adjusted *p* = 0.108).

### Post-COVID Assessment

Following discharge, one further patient in the non-strongly religious group has passed away. In addition, further patients were lost to follow-up. As a result, post-COVID-19 data were available in 21 non-highly religious and 58 highly religious patients. The post-COVID-19 assessment was carried out between 1 and 4 months after hospital discharge for the vast majority of patients.

There was no significant difference between the groups in clinical evolution and the occurrence of complications at the follow-up evaluation after the acute episode of SARS-CoV-2 infection. After adjusting for age, gender, vaccination status, and dexamethasone treatment, a significantly higher percentage of patients in the highly religious group had residual pulmonary changes compared to the non-highly religious group (Table 4). The majority of patients in both groups had ground-glass areas or reticular lesions at the follow-up CT scan.

**Table 4:** Post-COVID assessment

	Non-highly religious (*n* = 21)	Highly religious (*n* = 58)	*P* value*
Favorable clinical evolution (%)	21	13	0.70
Post-COVID-19 complication (%)	52	26	0.22
Post-COVID-19 pulmonary sequelae (%)	86	97	0.02
Ground-glass areas (%)	91	81	0.67
Reticular lesions (%)	52	72	0.06

*Adjusted for age, gender, vaccination status, radiological extension, and dexamethasone use

## Discussion

To our knowledge, this is the first study to evaluate the level of religiosity in a cohort of patients hospitalized for COVID-19 and to assess the correlations between religious beliefs and physical health outcomes.

According to the CRS-15 score, 97.3% of patients in our study were deemed as religious, while 68.7% were highly religious. These results are concordant with the latest data reported for Romanian general population during the 2021 census (Institutul Național de Statistică, 2023).

The previous research on gender differences in religiosity has shown that women tend to be more religious than men and tend to benefit more from what religion can offer in times of crisis (Blázquez & Sánchez-Mangas, 2023; Flere, 2007; Palmisano & Tadesco, 2019). Similarly, a number of studies have clearly demonstrated that mortality in SARS-CoV-2 infection is age and sex dependent, with higher mortality rates in males and older age (Nielsen et al., 2021; Torres et al., 2023).

We found no significant differences between highly religious and non-highly religious groups in age, gender, COVID-19 vaccination status, and prevalence of relevant comorbidities. Furthermore, patients in both groups displayed similar symptoms during their hospitalization for SARS-CoV-2 infection, had similar extension of radiological lung lesions, and there were no differences in the use of dexamethasone or antiviral treatment, the length of hospital stay, or ICU need. Despite these evidences of similar severity of disease in the two groups, we found significantly lower in-hospital mortality and significantly lower ferritin level suggestive of a less pronounced inflammatory response in the highly religious group.

After adjusting for age, gender, vaccination status, and dexamethasone treatment, the difference was no longer significant, but there was a trend toward lower mortality in the highly religious group. As there were no differences between groups in either of the adjustment factors, we believe that this result may be due to the relatively small sample size and warrants further testing in a larger population.

Several studies have assessed the impact of COVID-19 pandemic on mental health in various segments of the general population, including frontline health-care workers, and the role of religiosity as a resilience resource (Kantor & Kantor, 2020; Lucchetti et al., 2021). A systematic review of the studies measuring the impact of COVID-19 pandemic on mental health, as well as the effectiveness of religiosity as a coping mechanism found that in 80.77% of these studies, religiosity was associated with better mental health outcomes (Beaton et al., 2000).

However, few if any studies have assessed correlations between religiosity and physical health outcomes in the context of COVID-19 pandemic patients. In a study conducted on 731 Orthodox Jews, Pirutinsky et al. (2021) reported that the COVID-19 lockdown-related stress in this community was associated with weight gain for those with a low level of intrinsic religiosity, while for those with mean or higher intrinsic religiosity, stress and weight change were unrelated.

According to John Hopkins University, Baltimore, USA data, the case-fatality ratio in COVID-19 confirmed cases varied widely between reporting countries, ranging from 0.1 to 5.1% (2% in Romania) (Coronavirus Resource Center, 2022). These variations may be explained by differences in the number of people tested, demographics, or characteristics of the health-care systems. The Centers for Disease Control and Prevention (CDC) data from selected US hospitals show a mortality rate for hospitalized COVID-19 patients ranging from 2% (May 2022) to 24.1% (April 2020) (National Center for Health Statistics, 2022). In our cohort, in-hospital mortality rate was 5.3% for the whole study population, but with significant differences between highly religious and non-highly religious groups.

Since the early phases of COVID-19 pandemic, a number of studies have indicated the link between an exaggerated inflammatory response driven by the hyperproduction of proinflammatory cytokines and clinical evolution toward a fatal outcome (Costela-Ruiz et al., 2020; Huang et al., 2020; Zhang et al., 2020). Several biomarkers have been shown to correlate with the intensity of the inflammatory response in COVID-19: CRP, procalcitonin, IL-6, or serum ferritin. A meta-analysis including 16 studies, comprising 3962 patients with COVID-19, demonstrated the association between a high level of inflammatory markers on the one side and the severity of the disease and poor prognosis on the other side (Zeng et al., 2020). Furthermore, another observational study on 142 patients hospitalized with COVID-19 reported a significant lower level of serum ferritin in survivors compared to non-survivors (Parimoo et al., 2021). Similarly, in our study, the lower rate of mortality observed in the highly religious group correlated with a significantly lower level of serum ferritin in the same group.

A significant proportion of patients in our study population were found to have post-COVID-19 pulmonary sequelae at the follow-up evaluation. This may be first explained by the severity of the disease in this cohort of hospitalized patients, and second by the early date at which the follow-up assessment was carried out. We found a significantly higher prevalence of pulmonary sequelae in the highly religious group, which probably correlates with an improved survival rate. In a Chinese study conducted on a cohort of 1733 patients confirmed with SARS-CoV-2 infection, who underwent a post-COVID-19 follow-up evaluation at 6 months, the authors reported that more than 50% of the study participants had at least one abnormal CT pattern, regardless of the respiratory support required during their hospital stay (Huang et al., 2021).

Research in the field of religiosity and health has seen growing interest in recent decades, as reflected by an increasing number of publications (Koenig et al., 2021; Lucchetti & Lucchetti, 2014). Florence Nightingale, the founder of modern nursing, considered spirituality as an intrinsic component of human nature and “the most profound resource and powerful healing power available to the person” (Diego-Cordero et al., 2022). Although the previous research has analyzed the role of spirituality/religiosity as a coping mechanism in other respiratory conditions such as asthma, emphysema, or COPD (Adeli et al., 2014; da Silva et al., 2018; Green et al., 2011), or in debilitating conditions like chronic pain (Büssing et al., 2009), or conditions with a fatal outcome such as advanced cancer (Tarakeshwar et al., 2006), our study is probably the first to assess correlations between religiosity level as measured by a validated tool and physical health outcomes in patients hospitalized for COVID-19.

Although research about the link between religion and medicine remains controversial for the scientific community, the dismissive attitude per primam is not so generalized at present times (Levin, 2020). Results of scientifically verified studies demonstrating correlations between intrinsic religiosity and clinical evolution of patients with chronic or acute conditions, especially during general social crises, could raise awareness for health-care providers and physicians about the value of religious coping mechanisms to overcome physical and mental health problems, with the ultimate goal of improving patient well-being. Probably the best attitude regarding the role of religious and spiritual beliefs and practices in our patients’ care is best described by the words of psychiatrist and Holocaust survivor, Victor Frankl: “When a patient stands on the firm ground of religious belief, there can be no objection to making use of the therapeutic effect of his religious conviction and thereby drawing on his spiritual resources” (Frankl, 1959).

## Limitations

Our study has a number of limitations. First, the quantity of study participants is relatively small, warranting further prospective research with larger cohorts. Second, because some of the patients were critically ill and were not able to complete the religiosity questionnaire, we may have missed some useful data. Third, the follow-up data were not collected for all patients at a uniform interval after the acute episode of SARS-CoV-2 infection. Forth, religiosity was assessed at different time points, and some might have changed religiosity level depending on the outcome of their hospitalization for COVID-19.

## Conclusion

Religiosity may be an important resilience resource in times of disasters such as a global pandemic. The complexity of human nature warrants a multidisciplinary approach in order to deliver a scientifically sound research project regarding this controversial topic. We have highlighted in the current study that the level of religiosity in hospitalized patients with SARS-CoV-2 infection may correlate with health outcomes such as survival, level of inflammation, and the presence of pulmonary sequelae.

## References

[CR1] Adeli, S. H., Sekine, M. S., Fatemeh, H., & Mostafa, V. A. (2014). General health and religious coping strategies in patients suffering from Asthma. *Health, Spirituality and Medical Ethics,**1*(3), 2–9.

[CR2] Beaton, D. E., Bombardier, C., Guillemin, F., & Ferraz, M. B. (2000). Guidelines for the process of cross-cultural adaptation of self-report measures. *Spine,**25*(24), 3186. 11124735 10.1097/00007632-200012150-00014

[CR3] Blázquez, M., & Sánchez-Mangas, R. (2023). General and COVID19-specific emotional stress: Religious practice as a potential coping strategy. *Economics and Human Biology,**51*(101284), 101284. 10.1016/j.ehb.2023.101284 37531911 10.1016/j.ehb.2023.101284

[CR4] Büssing, A., Michalsen, A., Balzat, H. J., Grünther, R. A., Ostermann, T., Neugebauer, E. A., & Matthiessen, P. F. (2009). Are spirituality and religiosity resources for patients with chronic pain conditions? *Pain Medicine,**10*(2), 327–339. 10.1111/j.1526-4637.2009.00572.x 19284487 10.1111/j.1526-4637.2009.00572.x

[CR5] Chirico, F., & Nucera, G. (2020). An Italian experience of spirituality from the Coronavirus pandemic. *Journal of Religion and Health,**59*(5), 2193–2195. 10.1007/s10943-020-01036-1 32424660 10.1007/s10943-020-01036-1 PMC7233189

[CR6] Coronavirus resource center, Johns Hopkins University. 2022. Retrieved December, 2022 (https://coronavirus.jhu.edu)

[CR7] Costela-Ruiz, V. J., Illescas-Montes, R., Puerta-Puerta, J. M., Ruiz, C., & Melguizo-Rodríguez, L. (2020). SARS-CoV-2 infection: The role of cytokines in COVID-19 disease. *Cytokine & Growth Factor Reviews,**54*, 62–75. 10.1016/j.cytogfr.2020.06.001 32513566 10.1016/j.cytogfr.2020.06.001 PMC7265853

[CR8] da Silva, G. P., Nascimento, F. A., Macedo, T. P., Morano, M. T., Mesquita, R., & Pereira, E. D. (2018). Religious coping and religiosity in patients with COPD following pulmonary rehabilitation. *International Journal of Chronic Obstructive Pulmonary Disease,**13*, 175–181. 10.2147/copd.s146400 29379282 10.2147/COPD.S146400 PMC5757498

[CR9] de Diego-Cordero, R., Ávila-Mantilla, A., Vega-Escaño, J., Lucchetti, G., & Badanta, B. (2022). The role of spirituality and religiosity in healthcare during the COVID-19 pandemic: An integrative review of the scientific literature. *Journal of Religion and Health,**61*(3), 2168–2197. 10.1007/s10943-022-01549-x 35348988 10.1007/s10943-022-01549-x PMC8960681

[CR10] Dunn, W., Brown, C., & McGuigan, A. (1994). The Ecology of Human Performance: A framework for considering the effect of context. *The American Journal of Occupational Therapy: Official Publication of the American Occupational Therapy Association,**48*(7), 595–607. 10.5014/ajot.48.7.595 7943149 10.5014/ajot.48.7.595

[CR11] Flere, S. (2007). Gender and religious orientation. *Social Compass,**54*(2), 239–253. 10.1177/0037768607077035

[CR12] Frankl, V. (1959). *Man’s search for meaning*. Washington Square Press.

[CR13] Frent, S., Frent, M., & Popovici, A. (2022). Religiosity and mental health consequences of COVID-19 pandemic. *10.01—Respiratory Infections and Bronchiectasis.,**60*, 4280. 10.1183/13993003.congress-2022.4280

[CR15] Green, M. R., Emery, C. F., Kozora, E., Diaz, P. T., & Make, B. J. (2011). Religious and spiritual coping and quality of life among patients with emphysema in the national emphysema treatment trial. *Respiratory Care,**56*(10), 1514–1521. 10.4187/respcare.01105 21513606 10.4187/respcare.01105 PMC3630474

[CR16] Huang, C., Huang, L., Wang, Y., Li, X., Ren, L., Gu, X., Kang, L., Guo, L., Liu, M., Zhou, X., Lui, J., Huang, Z., Tu, S., Zhao, Y., Chen, L., Xu, D., Li, Y., Li, C., Peng, L., & Cao, B. (2021). 6-month consequences of COVID-19 in patients discharged from hospital: A cohort study. *The Lancet,**397*(10270), 220–232. 10.1016/S0140-6736(20)32656-8 10.1016/S0140-6736(20)32656-8 PMC7833295 33428867

[CR17] Huang, C., Wang, Y., Li, X., Ren, L., Zhao, J., Hu, Y., Zhang, L., Fan, G., Xu, J., Gu, X., Cheng, Z., Yu, T., Xia, J., Wei, Y., Wu, W., Xie, X., Yin, W., Li, H., Liu, M., & Cao, B. (2020). Clinical features of patients infected with 2019 novel coronavirus in Wuhan China. *The Lancet,**395*(10223), 497–506. 10.1016/S0140-6736(20)30183-5 10.1016/S0140-6736(20)30183-5 PMC7159299 31986264

[CR18] Huber, S., & Huber, O. W. (2012). The centrality of religiosity scale (CRS). *Religions,**3*(3), 710–724. 10.3390/rel3030710

[CR14] Huza, G. (2018). The psychometric properties of a Romanian version of the centrality of religiosity scale (CRS 15). *Religions,**10*(1), 11. 10.3390/rel10010011

[CR19] Institutul National de Statistica. (2023). *Rezultate definitive. Caracteristici etno-culturale demografice.* Retrieved November 6, 2023 from https://www.recensamantromania.ro/rezultate-rpl-2021/rezultate-definitive-caracteristici-etno-culturale-demografice/

[CR20] Kantor, B. N., & Kantor, J. (2020). Mental health outcomes and associations during the COVID-19 pandemic: A cross-sectional population-based study in the United States. *Frontiers in Psychiatry,**11*, 569083. 10.3389/fpsyt.2020.569083 33424655 10.3389/fpsyt.2020.569083 PMC7793873

[CR21] Koenig, H. G., Hamilton, J. B., & Doolittle, B. R. (2021). Training to conduct research on religion, spirituality and health: A commentary. *Journal of Religion and Health,**60*(3), 2178–2189. 10.1007/s10943-021-01193-x 33528715 10.1007/s10943-021-01193-x

[CR22] Levin, J. (2020). *Religion and medicine: a history of the encounter between humanity’s two greatest institutions*. Oxford University Press.

[CR23] Lucchetti, G., Góes, L. G., Amaral, S. G., Ganadjian, G. T., Andrade, I., Almeida, P. O. D. A., do Carmo, V. M., & Manso, M. E. G. (2021). Spirituality, religiosity and the mental health consequences of social isolation during Covid-19 pandemic. *International Journal of Social Psychiatry,**67*(6), 672–679. 10.1177/0020764020970996 33135559 10.1177/0020764020970996 PMC7649649

[CR24] Lucchetti, G., & Lucchetti, A. L. G. (2014). Spirituality, religion, and health: Over the last 15 years of field research (1999–2013). *International Journal of Psychiatry in Medicine,**48*(3), 199–215. 10.2190/pm.48.3.e 25492714 10.2190/PM.48.3.e

[CR25] Marshall, K., & Smith, S. (2015). Religion and Ebola: Learning from experience. *The Lancet,**386*(10005), e24–e25. 10.1016/S0140-6736(15)61082-0 10.1016/S0140-6736(15)61082-0 26159395

[CR26] National Center for Health Statistics. (2022). *In-hospital mortality among hospital confirmed COVID-19 encounters by week from selected hospitals*. Retrieved December 5, 2022, from https://www.cdc.gov/nchs/covid19/nhcs/hospital-mortality-by-week.htm

[CR27] Nielsen, J., Sarah, K., Nørgaard, S. K., Giampaolo, L. G., Lasse, S., Vestergaard, L. S., & Moelbak, K. (2021). Sex-differences in COVID-19 associated excess mortality is not exceptional for the COVID-19 pandemic. *Scientific Reports,**11*, 20815. 10.1038/s41598-021-00213-w 34675280 10.1038/s41598-021-00213-w PMC8531278

[CR28] Palmisano, S., & Todesco, L. (2019). The gender gap in religiosity over time in Italy: Are men and women really becoming more similar? *Social Compass,**66*(4), 543–560. 10.1177/0037768619868613

[CR29] Parimoo, A., Biswas, A., Baitha, U., Gupta, G., Pandey, S., Ranjan, P., Gupta, V., Barman Roy, D., Prakash, B., & Wig, N. (2021). Dynamics of inflammatory markers in predicting mortality in COVID-19. *Cureus*. 10.7759/cureus.19080 34868744 10.7759/cureus.19080 PMC8629097

[CR30] Pirutinsky, S., Cherniak, A. D., & Rosmarin, D. H. (2021). COVID-19, religious coping, and weight change in the orthodox Jewish community. *Journal of Religion and Health,**60*(2), 646–653. 10.1007/s10943-021-01196-8 33611679 10.1007/s10943-021-01196-8 PMC7897356

[CR31] Smith, B. W., Kay, V. S., Hoyt, T. V., & Bernard, M. L. (2009). Predicting the anticipated emotional and behavioral responses to an avian flu outbreak. *American Journal of Infection Control,**37*(5), 371–380. 10.1016/j.ajic.2008.08.007 19121548 10.1016/j.ajic.2008.08.007 PMC7124230

[CR32] Tarakeshwar, N., Vanderwerker, L. C., Paulk, E., Pearce, M. J., Kasl, S. V., & Prigerson, H. G. (2006). Religious coping is associated with the quality of life of patients with advanced cancer. *Journal of Palliative Medicine,**9*(3), 646–657. 10.1089/jpm.2006.9.646 16752970 10.1089/jpm.2006.9.646 PMC2504357

[CR33] Torres, C., García, J., Meslé, F., Barbieri, M., Bonnet, F., Camarda, C. G., Cambois, E., Caporali, A., Couppié, É., Poniakina, S., & Robine, J. M. (2023). Identifying age-and sex-specific COVID-19 mortality trends over time in six countries. *International Journal of Infectious Diseases,**128*, 32–40. 10.1016/j.ijid.2022.12.004 36509336 10.1016/j.ijid.2022.12.004 PMC9733967

[CR34] Zeng, F., Huang, Y., Guo, Y., Yin, M., Chen, X., Xiao, L., & Deng, G. (2020). Association of inflammatory markers with the severity of COVID-19: A meta-analysis. *International Journal of Infectious Diseases: IJID: Official Publication of the International Society for Infectious Diseases,**96*, 467–474. 10.1016/j.ijid.2020.05.055 32425643 10.1016/j.ijid.2020.05.055 PMC7233226

[CR35] Zhang, W., Zhao, Y., Zhang, F., Wang, Q., Li, T., Liu, Z., Wang, J., Qin, Y., Zhang, X., Yan, X., Zeng, X., & Zhang, S. (2020). The use of anti-inflammatory drugs in the treatment of people with severe coronavirus disease 2019 (COVID-19): The Perspectives of clinical immunologists from China. *Clinical Immunology,**214*, 108393. 10.1016/j.clim.2020.108393 32222466 10.1016/j.clim.2020.108393 PMC7102614

